# Aspects épidémiologiques, diagnostiques et thérapeutiques des ostéosarcomes de l'enfant au CHU Aristide le Dantec de Dakar: à propos de 16 cas

**DOI:** 10.11604/pamj.2013.14.104.1285

**Published:** 2013-03-16

**Authors:** Oumar Ndour, Desire Munyali Alumeti, Mbaye Fall, Aimée Faye Fall, Cheikh Diouf, Ndeye Aby Ndoye, Gabriel Ngom, Mamadou Ndoye

**Affiliations:** 1 Service de Chirurgie Pédiatrique CHU Aristide Le Dantec, Dakar, Sénégal

**Keywords:** Ostéosarcome, enfant, retard diagnostique, amputation, osteosarcoma, child, diagnostic delay, amputation

## Abstract

Le but de cette étude était de décrire les aspects épidémiologiques, diagnostiques et thérapeutiques des ostéosarcomes de l'enfant. Il s'agissait d'une étude rétrospective sur dix ans qui a colligé 16 dossiers d'ostéosarcome pris en charge au service de Chirurgie Pédiatrique de l'hôpital Aristide Le Dantec de Dakar. Les paramètres étudiés étaient le niveau d'instruction et le niveau socioprofessionnel des parents, l'origine géographique, l’âge, le sexe, les antécédents particuliers, le délai de consultation, les motifs de consultation, les signes physiques, les signes radiologiques, la biologie, les modalités thérapeutiques et l’évolution. Tous les patients avaient bénéficié d'un examen anatomopathologique qui a confirmé le diagnostic d'ostéosarcome. Pour la majeure partie de nos patients (58% des cas) les parents avaient un niveau d'instruction bas. L’âge moyen était de 11ans. Une prédominance masculine était retrouvée avec un sex-ratio de 3,25:1. Le délai de consultation moyen était de 16 mois. Le principal motif de consultation était la tuméfaction (10 cas). Huit patients avaient bénéficié d'un traitement traditionnel. La taille de la tumeur était supérieure à 10cm dans 14 cas. La localisation la plus fréquente était le genou (14 cas). La radiographie standard retrouvait dans 15 cas des images d'ostéolyse. Le bilan d'extension n'avait pas retrouvé de métastases. Les options thérapeutiques étaient dominées par l'amputation seule (43,75% des cas). La survie à 2 ans était de 17%. L'ostéosarcome atteint le plus souvent le garçon après l’âge de 10 ans. Sa prise en charge au Sénégal se heurte à d’énormes difficultés liées au retard diagnostique. La solution repose essentiellement sur une collaboration pluridisciplinaire.

## Introduction

L'ostéosarcome est la tumeur osseuse maligne primitive la plus fréquente chez l'enfant [1]. Il touche surtout le garçon avec un pic de fréquence entre 10 et 20 ans [2]. La survie a été complètement modifiée grâce à un diagnostic précoce et à la polychimiothérapie [3]. La réduction tumorale qu'elle induit rend possible la préservation du membre aux moyens de techniques de chirurgie conservatrice parfois complexes tout en assurant une résection carcinologiquement satisfaisante. Cette prise en charge permet aujourd'hui d'obtenir des taux de survie de près de 70% à 5 ans [1]. Cependant dans les pays en voie de développement comme le nôtre la prise en charge est confrontée à de nombreux problèmes avec au premier plan le retard diagnostique qui impose souvent des amputations de membres. Le but de notre étude était de décrire les aspects épidémiologiques, diagnostiques et thérapeutiques des ostéosarcomes de l'enfant au CHU Aristide Le Dantec de Dakar.

## Méthodes

Notre travail est une étude rétrospective menée de janvier 2001 à Décembre 2010, soit sur une période de dix ans. Elle comportait l'analyse de 16 dossiers d'enfants atteints d'ostéosarcome et pris en charge au service de Chirurgie Pédiatrique de l'hôpital Aristide Le Dantec de Dakar. Pour chaque dossier, les paramètres étudiés étaient le niveau d'instruction et le niveau socioprofessionnel des parents, l'origine géographique, l’âge, le sexe, les antécédents particuliers, le délai de consultation, les motifs de consultation, les signes physiques, les signes radiologiques (la radiographie standard, l’échographie, la tomodensitométrie), la biologie (Numération et formule sanguine), les modalités thérapeutiques (la chimiothérapie, la chirurgie, la radiothérapie) et l’évolution. Tous les patients ont bénéficié d'un examen anatomopathologique dont 6 sur pièces biopsiques et 10 sur pièces opératoires, qui ont confirmé dans tous les cas le diagnostic d'ostéosarcome. Les données ont été traitées sur Microsoft office excel.

## Résultats

**Niveau d'instruction et le niveau socioprofessionnel des parents:** Dans 58% des cas les parents (papa et maman) n’étaient pas scolarisés. Dans 35% des cas les parents avaient le niveau de l’école primaire. Chez un seul de nos patients, le père a fait des études supérieures (université). L'activité professionnelle des pères se répartissait comme suit: ouvrier: 08 cas; cultivateur: 07 cas; instituteur: 01 cas

**L'origine géographique:** La majeure partie de nos patients (12 cas soit 75%) provenait de la banlieue de Dakar. Le reste de nos patients provenait de diverses régions de l'intérieur du pays.

**Figure 1:**
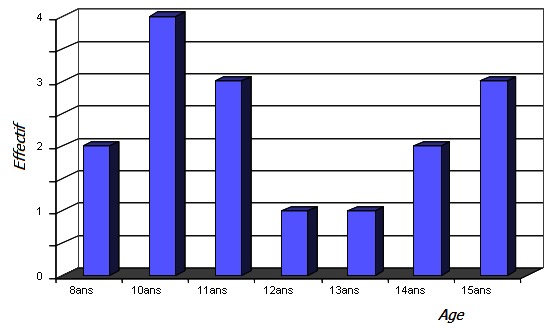
Répartition selon l’âge

**L’âge:** L’âge moyen de nos patients était de 11 ans avec des extrêmes de 8 ans et 15 ans. La Figure 1 montre la répartition selon l’âge.

**Le sexe:** Une prédominance masculine a été retrouvée avec un sex-ratio de 3,25.

**Les antécédents particuliers:** Deux de nos patients présentaient des antécédents de fracture 1 et 2 mois auparavant sur le siège même de la tumeur. Huit patients avaient bénéficié d'un traitement traditionnel. Sept patients avaient bénéficié d'une prise en charge dans une structure sanitaire avant d’être orienté vers le centre hospitalier de référence.

**Figure 2:**
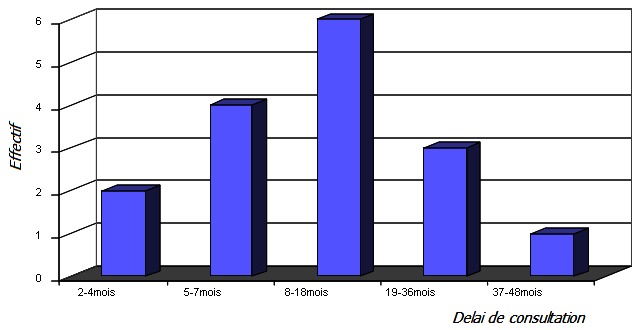
Répartition selon le délai de consultation

**Le délai de consultation:** Le délai écoulé entre le début de la symptomatologie et la première consultation à l'hôpital était en moyenne de 16 mois avec des extrêmes de 2 mois et 4 ans (Figure 2).

**Figure 3:**
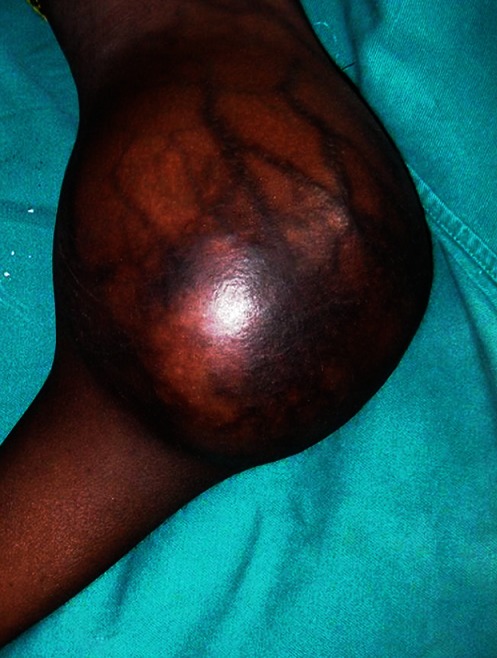
Ostéosarcome du genou de 20cm de diamètre de grand axe

**Les motifs de consultation:** Pour la majeure partie des patients (10 cas soit 62,5%) le motif de consultation a été une tuméfaction (Figure 3). Deux patients étaient venus en consultation pour un traumatisme. Dix patients ont consulté pour une impotence fonctionnelle dont 8 relatives et 2 absolues. Trois patients étaient adressés par une structure sanitaire périphérique et un patient par un hôpital régional pour une meilleure prise en charge d'une tumeur.

**Les signes physiques:** Une altération de l’état général avec asthénie, amaigrissement et anorexie était retrouvée chez 7 patients. La douleur était présente dans tous les cas. Une boiterie a été notée dans 10 cas. La taille de la tumeur a été mesurée selon son grand axe. Elle était supérieure à 10cm dans 14 cas et inférieure à 10cm dans 2 cas. Les localisations étaient variables (Tableau 1). Des marques de scarification cutanée en regard de la tuméfaction ont été retrouvées dans 8 cas. Des adénopathies inguinales homolatérales à la tuméfaction ont été retrouvées dans 10 cas.

**Tableau 1:** Répartition selon la topographie

Siège	Nombre	Pourcentage
Genou	14	87,5%
Hanche	1	6,25%
Epaule	1	6,25%
**Total**	**16**	**100%**

**Les signes radiologiques:** Une radiographie standard du membre atteint avec des clichés de face et de profil était réalisée chez tous les patients. Elle retrouvait dans 15 cas des images d'ostéolyse, une image lacunaire dans 1 cas et des appositions périostées dans 7 cas. n patient a présenté une fracture pathologique. Le Tableau 2 montre les différentes localisations sur la radiographie standard. Dans le cadre du bilan d'extension de la tumeur, 5 patients avaient bénéficié d'une radiographie du thorax de face, 1 d'une tomodensitométrie et 2 d'une échographie abdomino-pelvienne. Ce bilan d'extension n'avait pas retrouvé de métastases.

**Tableau 2:** Répartition des images radiographiques selon la topographie

Siége	Nombre	Pourcentage
Extrémité supérieure de l'humérus	1	6,25%
Extrémité supérieure du fémur	1	6,25%
Extrémité inférieure du fémur	13	81,25%
Extrémité supérieure du tibia	1	6,25%
**Total**	**16**	**100%**

**La biologie:** La numération de la formule sanguine retrouvait une anémie dans 15cas avec une moyenne de 7 g/dl.

**Les modalités thérapeutiques:** Les différentes options thérapeutiques sont résumées sur le Tableau 3.

**Tableau 3:** Répartition des patients selon les options thérapeutiques

Traitement	Nombre	Pourcentage
Amputation seule	7	43,75%
Amputation puis chimiothérapie	6	37,5%
Chimiothérapie puis amputation	1	6,25%
Chimiothérapie puis amputation et ensuite chimiothérapie	1	6,25%
Chimiothérapie seule	1	6,25%
**Total**	**16**	**100%**

**L’évolution:** Les suites thérapeutiques étaient évaluées après un recul moyen de 14 mois avec des extrêmes de 2 mois et 2 ans. Trois cas de métastases ont été notés dont 1 pelvienne et 2 pulmonaires respectivement à 2 mois, 5 mois et 7 mois après amputation. La survie à 2 ans était de 17%.

## Discussion

### Aspects épidémiologiques

Les tumeurs osseuses malignes de l'enfant restent une entité fort heureusement assez rare, puisqu'elles ne représentent que 5% de toutes les tumeurs malignes pédiatriques [1].L'ostéosarcome est la plus fréquente des tumeurs malignes du squelette chez l'enfant. Il représente 15 à 35% des tumeurs malignes primitives de l'os [2]. Dans notre étude pendant la même période 69 tumeurs malignes primitives en dehors de l'ostéosarcome ont été répertoriées soit un chiffre de 18,82% pour les ostéosarcomes.

L'ostéosarcome survient chez l'enfant entre 10 et 20 ans. Selon Parkin [4] 80% des ostéosarcomes surviennent chez l'enfant et l'adolescent avec un âge moyen de survenue de 14 ans. Il a montré par ailleurs que la fréquence diminuait avec l’âge. D'exceptionnels cas ont été rapportés avant 5ans. Selon Philip [5] l’âge inférieur à 12 ans est un facteur de mauvais pronostic. Dans notre étude l’âge était compris entre 8 et 15 ans avec une moyenne de 11 ans. Une prédominance masculine est rapportée dans la plupart des études épidémiologiques avec un sex-ratio tournant autour de 1,7 [6, 7]. Dans notre étude la prédominance masculine est plus nette avec un sex ratio de 3,25.

Les localisations préférentielles des ostéosarcomes sont le genou, l’épaule et la hanche [7, 8]. Dans notre étude la localisation la plus fréquente était le genou (87,5% des cas) avec au premier plan l'extrémité inférieure du fémur (81,25%). Dans les séries d'Arndt [9], Campanacci et Widhe [10] qui ont colligé au total 3433 cas, les atteintes du genou représentaient plus de la moitié des cas (53%) suivies de l'humérus (10%).

Les ostéosarcomes ont souvent un potentiel de malignité élevé avec une évolution fulminante. C'est dire que tout point d'appel tumoral au niveau de l'appareil locomoteur doit être examiné et pris en charge en tenant compte de cet aspect afin d’éviter tout retard diagnostique et thérapeutique. Si dans les pays développés des avancées massives ont été faites dans la prise en charge précoce, dans les pays en voie de développement comme le nôtre tel n'est pas le cas. En effet le premier niveau de difficulté est le retard diagnostique qui a une forte répercussion sur la prise en charge thérapeutique. Dans notre étude nous avons retrouvé que chez 10 de nos patients soit 62,5% des cas, le délai de consultation était supérieur à 7 mois. Ce retard de consultation s'explique à plusieurs niveaux. Dans notre série les parents avaient un niveau d'instruction bas et nos considérations traditionnelles font que toute tuméfaction est prise à tort comme du sang coagulé et le premier réflexe est d'aller voir un tradipraticien pour évacuer ce sang. Nous avons retrouvé que 8 patients avaient bénéficié d'un traitement traditionnel confirmé par les marques de scarification retrouvées à l'examen physique. Il faut insister sur le danger de ce traitement traditionnel. En effet ces scarifications consistent à faire de petites mouchetures cutanées pour ainsi pouvoir aspirer du sang et il a été démontré tout le risque que constitue le saignement car ce dernier favorise la diffusion tumorale [1]. Le croisement entre le délai de consultation et les patients ayant bénéficié d'un traitement traditionnel a montré que chez les 10 patients qui ont présenté un délai de consultation supérieur à 7 mois, huit avaient bénéficié d'un traitement traditionnel.

Le deuxième volet est la prise en charge au niveau des structures sanitaires périphériques. En effet lorsque le traitement traditionnel ne marche pas, secondairement les parents s'orientent souvent vers la structure sanitaire la plus proche. Dans notre étude nous avons trouvé que tous nos patients provenaient soit de la banlieue soit de l'intérieur du pays et avaient bénéficié d'une prise en charge au niveau d'une structure sanitaire inadéquate. A ce niveau la tuméfaction est souvent prise à tort comme une simple inflammation ou une infection et une radiographie n'est pas toujours systématiquement demandée ou n'est pas toujours accessible.

### Aspects diagnostiques

Les patients sont souvent adressés vers la structure hospitalière de référence devant l'augmentation importante de volume de la tuméfaction ou devant l'altération de l’état général. Les différents facteurs cités plus haut font que la majeure partie de nos patients sont vus avec une masse tumorale importante et un état général souvent altéré rendant davantage difficile la prise en charge en milieu hospitalier. Les modes de découverte et les motifs de consultation sont nombreux, sources assez souvent de retard diagnostique en raison de l'absence de spécificité de signes cliniques et de la rareté de cette pathologie. Ainsi le diagnostic est rarement évoqué en première intention. Cependant dans notre étude devant le volume souvent important de la masse tumorale (taille > 10cm dans 87,5% des cas) et la rapidité d’évolution, le diagnostic a été souvent évoqué. Le volume tumoral a également un intérêt pronostic. En effet selon Philip [5] une taille supérieure à 10cm est un facteur de mauvais pronostic. Devant l'absence de spécificités cliniques, des règles de prise en charge ont été définies [11–13]. La mise en route des investigations complémentaires est une véritable urgence, puisque de sa rapidité dépend la date de réalisation de la biopsie, et surtout l'analyse histologique. Les examens radiographiques visent à préciser l'extension locale et générale. Les aspects radiologiques évocateurs d'ostéosarcome ne sont pas forcément spécifiques. Certes une lyse osseuse hétérogène, des appositions périostées en pelure d'oignon [2] à fortiori des images en feu d'herbe, ou une rupture corticale sont très suspects d’être en rapport avec un processus malin. Cependant ils témoignent simplement d'une évolution rapide et agressive pour l'os de la pathologie en cause, sans pour autant préjuger de son étiologie. Dans notre étude le retard à la consultation constitue un facteur favorable au développement de la tumeur. C'est pourquoi les images radiographiques étaient très évocatrices (ostéolyse importante avec apposition périostée) au stade du diagnostic. Un bilan d'extension est systématique avant tout geste chirurgical y compris biopsique [11, 12]. Il repose sur la radiographie pulmonaire, la tomodensitométrie, la scintigraphie osseuse et l'imagerie par résonance magnétique nucléaire [13]. Cependant ces trois derniers examens d'acquisition récente ne sont pas toujours accessibles dans notre contexte et ont un coût élevé. C'est pourquoi un seul de nos patients a pu bénéficier d'une tomodensitométrie qui n'avait pas retrouvé de métastases. Cette difficulté d'acquisition de ces examens complémentaires rend la prise en charge aléatoire. Ce bilan d'extension ne doit pas cependant retarder la biopsie. En revanche il doit impérativement précéder la mise en route du traitement, en raison du pourcentage assez élevé de tumeurs déjà métastatiques au diagnostic [1]. La biopsie est la véritable clé diagnostique et est systématique. Cependant dans notre contexte d'exercice il n'est pas toujours réalisable pour diverses raisons. Devant un délai de consultation très long ayant permis à la tumeur d'avoir tout le temps de s'exprimer, avec un état général altéré et des images radiographiques évidentes faudrait-il toujours réaliser une biopsie et attendre les résultats, au risque de s'exposer à des métastases ‘ En effet dans notre contexte pour avoir les résultats anatomopathologiques il faut au minimum un mois après la biopsie.

### Aspects thérapeutiques

Les modalités du traitement sont aujourd'hui parfaitement codifiés, et font appel à des protocoles nationaux voire européens [11, 13, 14]. Les stratégies du traitement associent une chimiothérapie pré opératoire, suivie d'une résection tumorale, puis d'une chimiothérapie post opératoire qui varie en fonction du pourcentage de cellules vivantes résiduelles sur la pièce de résection [1]. Ce protocole n'a pu être réalisé dans notre étude que chez un seul patient. La plupart ayant bénéficié soit d'une amputation seule, soit d'une chimiothérapie suivie d'une amputation. Ce problème est lié au fait que le protocole thérapeutique est lourd sur le plan économique mais également sur le plan de la durée. Actuellement l'existence d'un programme d'aide a nettement amélioré le volet économique. Cette chimiothérapie nécessite également que le patient ait un état général acceptable pour pouvoir la supporter mais également un bilan pré chimiothérapie qui élève davantage le coût de la prise en charge et parfois même retarde le début du traitement. C'est pourquoi dans notre contexte d'exercice notre première arme thérapeutique est l'amputation d'emblée. Si l'amputation a longtemps été la seule attitude curative dans le traitement de l'ostéosarcome, ces 20 dernières années ont vu un essor considérable de la chirurgie conservatrice grâce à l'amélioration des indications chirurgicales et des procédés de reconstruction reposant sur des prothèses [12, 15]. Cette chirurgie conservatrice est estimée actuellement à environ 80% [3]. Cependant dans notre contexte d'exercice elle n'est pas encore de mise car faudrait-il encore que le diagnostic puisse être posé précocement et que le matériel prothétique soit disponible, ce qui n'est pas le cas. Ceci nous pousse souvent à un geste mutilant chez un enfant en pleine croissance. Ce geste chirurgical lourd retentit de façon significative sur l’état général de l'enfant et réduit les chances d'une meilleure tolérance à la chimiothérapie adjuvante. Dans notre étude un patient n'a pas pu bénéficier d'un traitement chirurgical. Ce dernier n'a bénéficié que d'une chimiothérapie néo adjuvante qui a été mal tolérée. La place de la radiothérapie dans le traitement de l'ostéosarcome a été précisée dans un ‘‘Standard Option, Recommandation’’ publié en 2005 [16]. Cette radiothérapie est de principe non indiquée dans le traitement de première intention d'un ostéosarcome, excepté en cas de tumeur non accessible à la chirurgie, de refus de la chirurgie ou de résection marginale. Dans notre série il n'y a pas eu d'indication à cette radiothérapie.

Le but du traitement est d'améliorer la qualité de vie de l'enfant mais également sa survie. Actuellement avec l'introduction de nouvelles méthodes thérapeutiques, le taux global de survie à 5 ans est d'environ 80% [1]. Dans notre série la survie à 2 ans était de 17%. Le taux de décès précoce (moins de 2 ans) était généralement élevé (41% des cas). Ces décès étaient liés soit à une mauvaise tolérance de la poly chimiothérapie ou à la chirurgie elle-même, soit à la survenue de métastases surtout pulmonaires.

## Conclusion

L'ostéosarcome de l'enfant est une pathologie qui atteint souvent le garçon après l’âge de 10 ans. Elle se localise préférentiellement au niveau de l'extrémité inférieure du fémur. Sa prise en charge rencontre des difficultés considérables au Sénégal du fait du retard diagnostique, de l'insuffisance du plateau technique et du bas niveau de vie des populations. L'amélioration de cette prise en charge doit passer par un certain nombre de mesures: une campagne de sensibilisation et d'information doit être fait à 2 niveaux: la population elle-même mais également le personnel médical et paramédical des structures sanitaires périphériques et des régions, avec parfois même des programmes de formation pour la prise en charge; une collaboration pluridisciplinaire entre le chirurgien pédiatre, l'oncologue pédiatre, le radiologue et l'anatomopathologiste; une amélioration du plateau technique avec notamment l'acquisition de prothèses.
